# Competition between RSV and influenza: Limits of modelling inference from surveillance data

**DOI:** 10.1016/j.epidem.2021.100460

**Published:** 2021-06

**Authors:** Naomi R. Waterlow, Stefan Flasche, Amanda Minter, Rosalind M. Eggo

**Affiliations:** Department of Infectious Disease Epidemiology, London School of Hygiene & Tropical Medicine, UK

**Keywords:** Inference, Competition, Influenza, Respiratory syncytial virus, Interaction

## Abstract

Respiratory Syncytial Virus (RSV) and Influenza cause a large burden of disease. Evidence of their interaction via temporary cross-protection implies that prevention of one could inadvertently lead to an increase in the burden of the other. However, evidence for the public health impact of such interaction is sparse and largely derives from ecological analyses of peak shifts in surveillance data. To test the robustness of estimates of interaction parameters between RSV and Influenza from surveillance data we conducted a simulation and back-inference study. We developed a two-pathogen interaction model, parameterised to simulate RSV and Influenza epidemiology in the UK. Using the infection model in combination with a surveillance-like stochastic observation process we generated a range of possible RSV and Influenza trajectories and then used Markov Chain Monte Carlo (MCMC) methods to back-infer parameters including those describing competition. We find that in most scenarios both the strength and duration of RSV and Influenza interaction could be estimated from the simulated surveillance data reasonably well. However, the robustness of inference declined towards the extremes of the plausible parameter ranges, with misleading results. It was for instance not possible to tell the difference between low/moderate interaction and no interaction. In conclusion, our results illustrate that in a plausible parameter range, the strength of RSV and Influenza interaction can be estimated from a single season of high-quality surveillance data but also highlights the importance to test parameter identifiability *a priori* in such situations.

## Highlights

• Respiratory Syncytial Virus (RSV) and Influenza may interact through innate immunity.
• Interaction can result in short-term cross-protection.
• Mathematical models can be used to identify cross-protection from surveillance data.
• Model inference must accurately and precisely identify parameter values.
• The UK respiratory season can be used as a case study to evaluate back-inference.

## 1 Introduction

Respiratory Syncytial Viruses (RSV) and seasonal influenza viruses cause large burdens of respiratory disease, including in young children (Lafond et al., 2016; Shi et al., 2017). RSV was recently identified as the primary cause of hospitalisation for severe paediatric pneumonia (Pneumonia Etiology Research for Child Health (PERCH) Study Group, K. L. et al., 2019), particularly in the neonatal period. In the northern hemisphere both viruses cause pronounced annual winter epidemics peaking between October and March (Bloom-Feshbach et al., 2013).

Evidence from epidemiological and biological studies implies there is competitive interaction between influenza and RSV (Opatowski et al., 2017; Velasco-Hernández et al., 2015; Mak et al., 2012; Pascalis et al., 2012; Yang et al., 2012; Walzl et al., 2000). The biological mechanism for competition is activation of the innate “antiviral response” by infection that can inhibit further or subsequent infection (Walzl et al., 2000; Ascough et al., 2018; Lee et al., 2018), resulting in a period of cross-protection during and after infection. Mouse studies have shown this effect, where following influenza infection or live attenuated influenza vaccination (LAIV), RSV replication/severity was decreased (Walzl et al., 2000; Lee et al., 2018). Within-host animal studies, both in vitro and modelling, have shown that the growth rates of the viruses can be affected by other viruses present (Pinky and Dobrovolny, 2016; Shinjoh et al., 2000). The duration of this cross-reactive response is debated, varying from “short-term” (Ferguson et al., 2005), less than two weeks (Laurie et al., 2015) or up to 3 months (Kelly et al., 2010). Influenza epidemics caused by different strains are thought to exhibit competitive exclusion (Opatowski et al., 2017; Ferguson et al., 2003; Kucharski et al., 2016), and for RSV and influenza syndromic surveillance has shown shifts in the seasonal incidence peaks of RSV following abnormal (pandemic or early) influenza seasons (Mak et al., 2012; Hirsh et al., 2014; Meningher et al., 2014; Gröndahl et al., 2014; Casalegno et al., 2010; van Asten et al., 2016), which suggest this mechanism may not only occur but can substantially alter the epidemiology of influenza and RSV. There is, however, little evidence that links the strength of competition between RSV and influenza within a host to observed population dynamics. Understanding the dynamics is critical for predicting the effects of alteration of their ecological balance, for example through vaccination programs, and is the motivation for this study.

Influenza vaccination rates, especially in key transmission groups could disrupt transmission, potentially leading to effects on interacting viruses. In the UK, the RSV epidemic usually precedes the influenza epidemic by one or two months, so reduced influenza transmission as a result of childhood influenza vaccination may not affect RSV transmission dynamics. However, the competitive pressure exhibited by RSV on influenza may become highly relevant soon. The only RSV vaccine candidate yet that completed Phase 3 trials, the maternal vaccine, Novavax, demonstrated only partial efficacy that the Advisory Committee for Immunization Practices in the US deemed insufficient to warrant licensure (Novavax Announces, 2021). However, the RSV vaccine pipeline contains a number of Phase 1 and 2 candidates that aim to protect children in part by limiting RSV circulation. As such, these future RSV vaccines have the potential to decrease the competitive pressure on influenza and thereby increase influenza as an unintended consequence, both in children and other age groups, as children are a key driver of transmission. These impacts will need to be considered as part of their cost benefit proposition preceding routine use.

Mathematical modelling is an important tool for testing mechanisms and hypotheses of epidemiologically significant RSV and influenza competition, such as the hypothesis that they competitively interact. Models offer an opportunity to mechanistically combine observations from surveillance data and extrapolate beyond the observed. However, in the case of RSV and influenza competition the identifiability of model parameters from viral surveillance data is uncertain. Hence, we conducted a simulation study to test whether parameters can be back-inferred from a range of realistic model-generated scenarios that include only partial observation of the infection dynamics from surveillance-like data.

## 2 Methods

### 2.1 Model structure

**Fig. 1:**
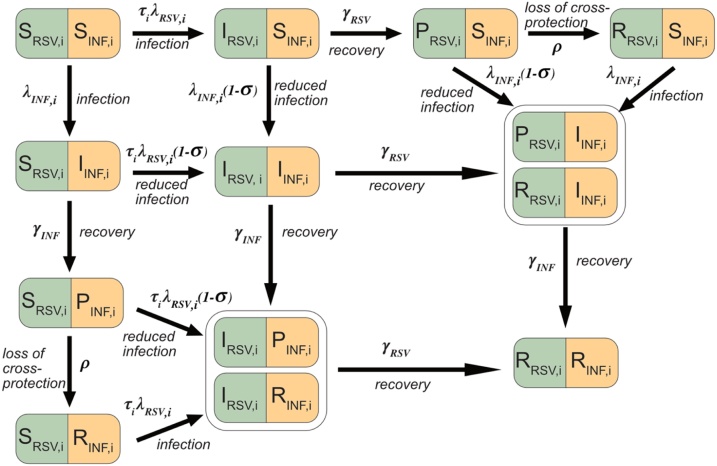
Model diagram for RSV and Influenza (INF). Individuals could be either Susceptible (S), Infected, (I), Protected (P) or Recovered (R) to either virus. Following infection, (which occurred at rate $\lambda_{RSV,i}$ and $\lambda_{INF,i}$), recovery occurred at a constant rate ($\gamma_{RSV}$ and $\gamma_{INF}$), and the population entered the P state. Here they are immune to the virus they were infected by and protected to a varying extent (σ) against infection from the second virus. This protection waned at rate ρ, and the population entered the R compartment. In the R compartment the population was immune to the virus it was infected by, but not the other virus. We ran the model for one season and compartments I_RSV,i_P_INF,i_ and I_RSV,i_R_INF,I,_ were combined, and P_RSV,i_I_INF,I_ and R_RSV,i_I_INF,I_ were combined, because they are effectively identical. Parameters were: age susceptibility to RSV infection ($\tau_{i}$), For clarity, age structure is given only by the subscript (i), for further details see supplement section 2.

We developed an age-stratified deterministic compartmental transmission model for RSV and Influenza with interactions (Fig. 1 and Supplement Section 2). The population could be Susceptible (*S*), Infectious (*I*), Protected (*P*) or Recovered (*R*) for each of RSV and influenza viruses. We simulated one season so we did not consider potential loss of immunity, and current estimates for RSV immunity lasts less than a year (Weber et al., 2001), and we take influenza immunity into account by fitting the percentage susceptible at the start of the season (see below). There were separate transmission and recovery rates for each virus (subscripts RSV and INF), and *i* and *j* denote age groups. Susceptible individuals were infected at rates $\lambda_{INF,\;i}\;$ and $\lambda_{RSV,\;i}$ and enter the *I* compartment. They recovered at rates $\gamma_{INF}$ and $\gamma_{RSV}$, and entered the *P* compartment where they were no longer infectious. In *P*, individuals were fully protected against homologous re-infection and also had some cross protection against the second virus. Loss of cross-protection occurred at rate *ρ*. Infection with a second virus was less likely in the *I* and *P* classes and occurred at a rate reduced by (σ). The key parameters determining interaction are therefore the strength of competition (σ) and the rate of loss of cross-protection (ρ). Compartments *I_RSV_,_i_P_INF,i_* and *I_RSV,i_R_INF,I_,* as well as *P_RSV,i_I_INF,I_* and *R_RSV,i_I_INF,I_* were combined as they were effectively identical when modelling only one season.

The model was stratified into 5 age categories: infants: 0−1 years, pre-school-aged children: 2−4 years, school-aged children: 5−15 years, adults: 16−64 years, and older adults: aged 65 + . Age-dependent contact patterns relevant to the transmission of infections are highly age heterogenous (Mossong et al., 2008), and we used social contact patterns (including both physical and verbal contacts) in England from POLYMOD (Mossong et al., 2008), a European wide contact study in 2005/6, and in the socialmixr R package (Funk, 2018). We calculated forces of Infection, $\;\lambda_{RSV,i}$ and $\lambda_{INF,i}$, from the baseline transmission rates $\beta_{INF}$ and $\beta_{RSV}$ and the mixing parameters as:

(1) $$\lambda_{RSV,\;i}={\;\sum_{j=1}^{5}\beta_{RSV}\alpha }_{ij}I_{RSV,j}$$

(2) $${\text{λ}}_{INF,i}={\;\sum_{j=1}^{5}\beta_{INF}\alpha }_{ij}I_{INF,j}$$

where $\alpha_{ij}$ is the contact rate between groups *i* and *j* and $I_{INF,j}$ and $I_{RSV,j}$ are the proportion infected with Influenza and RSV in age group *j*. See Supplement Section 1 for model equations.

We modelled one year from the start of the respiratory virus season, and initiated the model with a proportion of each age group susceptible to influenza set from serological data (Baguelin et al., 2013) (Table 2) and the rest in *S_RSV_R_INF_*. RSV immunity to re-infection may last less than a year (Weber et al., 2001), therefore we considered the population to be susceptible to RSV at the start of the season. However, RSV susceptibility differs with age (Henderson et al., 1979), and therefore we reduced the susceptibility to the same range as in other models (Moore et al., 2014) by decreasing the infection rate by a susceptibility parameter, $\tau_{i}$ (Table 2).

RSV was seeded at time $\eta_{RSV}$ when one individual from the fully Susceptible class (*S_RSV_S_INF_*) becomes infected (*I_RSV_S_INF_)*. Influenza infections are introduced at a rate of 0.1 cases per day from *S_RSV_S_INF_* to *S_RSV_I_INF_,* starting on day $\eta_{INF}$. Influenza introduction assumptions differ from those of RSV as with a single introduction the influenza epidemic was supressed for the whole season at certain parameter values, which is not seen in UK surveillance. See Supplementary section 6 for further details.

An observation process layer converted infections to detected cases using a binomial distribution. The number of detected cases is assumed to follow a binomial distribution as follows:

(3) $$P\left(x_{virus,i,t}=X\right)=\;\left(\begin{array}{c} n_{virus,i}\\ X_{virus,i} \end{array}\right){{\text{Δ}}_{virus,\;i}}^{X}({1-{\text{Δ}}_{virus,i})}^{\left(n_{virus,i}-X_{virus,i}\right)}$$

where X is the number of detected cases in n infections and $x_{virus,i,t}$ is the cases detected for each virus, age group, and timestep. The proportion detected was different for each age group and virus (Table 2).

We implemented the model in R (R Core Team, 2018) and C++ using the Rcpp (Eddelbuettel and Francois, 2011) and deSolve (Soetaert et al., 2010) packages.

### 2.2 Simulated data

We generated simulations to resemble data collected through surveillance systems in the UK (Reeves et al., 2017), such as the Respiratory Datamart System in England and Wales (Zhao et al., 2014) and other Public Health England surveillance systems (Weekly national flu reports, 2021; Respiratory infections, 2021). This provides laboratory test results from routinely tested clinical respiratory samples from a range of respiratory viruses. The proportion detected varies by age-group for RSV (Table 1), as younger infants are more likely to present with severe symptoms (Ohuma et al., 2012). Output from the model is weekly number of positive tests in the under-five population for RSV and influenza.

**Table 1:** Parameter values used for generating simulations.

Parameter	Symbol	Value used in simulations	Source	Status in inference
Duration of infectiousness for RSV	$1/\gamma_{RSV}$	9 days	Weber et al. (2001)	Fixed
			Range from published papers: 6.7−12 days (Weber et al., 2001; Moore et al., 2014; Hall et al., 1976)	
Transmission parameter for RSV	$\beta_{RSV}$	0.043	Calibrated to observed values. Equates to an *R*_0_ and *R_effective_* of 2.5. See Supplementary section 3 for details	Estimated Log scale
Time of first infection for RSV	$\eta_{RSV}$	Day 1	Calibrated to observed pattern. See Supplementary section 6 for details.	Estimated
Age susceptibility to RSV infection (0−1, 2−4, 5−15, 16−64, 65+)	$\tau_{i}$	1, 0.75, 0.65, 0.65, 0.65	Henderson et al. (1979) see supplement section 4.	Fixed
Proportion of RSV infections in ages 0−1 detected	${\text{Δ}}_{RSV1}$	0.004	Calibrated to observed values. See Supplementary section 5 for details.	Estimated
				Log odds scale
Proportion of RSV infections in ages 2−4 detected	${\text{Δ}}_{RSV2}$	0.001	Calibrated to observed values. See Supplementary section 5 for details	Estimated
				Log odds scale
Duration of infectiousness for influenza	$1/\gamma_{INF}$	3.8 days	Cauchemez et al. (2004)	Fixed
			Range from published papers: 1−4.5 days (Cauchemez et al., 2004; Hayden et al., 1999; Chowell et al., 2011; Cori et al., 2012)	
Transmission parameter for Influenza	$\beta_{INF}$	0.063	Calibrated to observed values. Equates to an *R*_0_ of 2.91, *R_effective_* of 1.55. See Supplementary section 3 for details.	Estimated
				Log scale
Time of first infection for Influenza	$\eta_{INF}$	Day 10	Calibrated to observed pattern. See Supplementary section 6 for details.	Estimated
Proportion susceptible to influenza (<2, 2−4, 5+)	$\omega_{i}$	1, 0.688, 0.525	Assuming born susceptible (Nokes et al., 2004), then values from Baguelin et al from serology data from 2003/4 (Baguelin et al., 2012)	Fixed
Proportion of Influenza infections in ages 0−4 detected	${\text{Δ}}_{INF}$	0.002	Calibrated to observed values. See Supplementary section 5.	Estimated
				Log odds scale
Strength of interaction	σ	0.01, 0.1, 0.2, 0.3, 0.4, 0.5, 0.6, 0.7, 0.8, 0.9, 0.99	Range of values tested	Estimated
Rate of loss of protection	ρ	0.025, 0.05, 0.1, 0.2, 0.5 per day	Range of values tested	Estimated
				Log scale

**Table 2:** Demography and susceptibility input used for model simulations.

Demography	Value used	References/Comments
Population size	56 758 452	UK. Demography from POLYMOD (Mossong et al., 2008)
Population 2−4 years	2070936	UK. Demography from POLYMOD (Mossong et al., 2008)
Population <2 years	1380624	UK. Demography from POLYMOD (Mossong et al., 2008)

We generated simulations with parameter values from the literature and if unavailable we calibrated the values to realistic ranges (Table 1). Across simulations we varied σ (strength of interaction), for which we used 11 different values, and ρ (the rate of loss of cross-protection), for which we used 5 different values. This resulted in 55 combinations of σ and ρ and we simulated 5 replicates of each.

### 2.3 Parameter estimation

We assumed that the observed cases followed a Poisson distribution with likelihood:

(4) $$L\left(\theta ,x_{1}\ldots \;x_{n}\right)=\;\sum_{j=1}^{n}e^{-\theta }\frac{1}{x_{j}!}\;\theta^{x_{j}}\;$$

where  θ is the modelled detected cases of RSV and influenza in the two youngest age groups, $x_{j}$ is the observation and n is the total number of observations. We fitted only to the lowest 2 age groups to represent where the majority of samples for RSV are taken from and detected. We fitted the model to simulated data using Metropolis Hastings Markov Chain Monte Carlo (MCMC) sampling. Estimated parameters were transmission rates ($\beta_{RSV}$, $\beta_{INF})\;$ detection probabilities (${\text{Δ}}_{R\text{S}\text{V}2}$, $\Delta_{INF}$) interaction parameters (ρ, σ) and season start times ($\eta_{RSV}$, $\eta_{INF}$). For each scenario, we ran two chains with 450 000 iterations as burn in followed by a further 250 000 iterations. For chains that did not converge, we extended the chains for a further 250 000 iterations iteratively until convergence was reached or a total of 1 200 000 iterations were run. We used weak priors, and the priors for $\beta_{RSV}$ and $\beta_{INF}$ were calculated from *R_0_* values, assuming no interaction (see Supplement Section 3). We adapted the shape of the proposal distribution during burn in, from 5000 accepted proposals to a further 300000 proposals, to take correlation between parameters into account by allowing the covariance matrix for proposal distributions to change. Parameter limits are defined in Supplement Section 7 and $\beta_{RSV}$, $\beta_{INF},$ ${\text{Δ}}_{RS\text{V}1}$, ${\text{Δ}}_{R\text{S}\text{V}2}$, $\Delta_{INF}$ and ρ were sampled on a log scale to improve mixing where the parameter values were very low.

We assessed MCMC convergence via the Gelman-Rubin statistic, which compares the within-chain variance to the between-chain variance for each parameter. Scenarios with a statistic greater than 1.1 we deemed as practically unidentifiable from simulated data. We also calculated the Pearson correlation coefficient between each two estimated parameters and assessed how these changed with the values of the interaction parameters (σ and *ρ*), in order to further understand difficulties with parameter estimation.

We compared the inferred parameter estimates to the simulated parameter values to determine inaccuracy and imprecision of the fit, where inaccuracy is defined as the difference between the median value of the posterior distribution and the true value, and imprecision is the range between the 95 % credible intervals (95 % CI). We present results from one replicate set of simulations in Results and others are given in the supplement.

## 3 Results

### 3.1 Epidemic profiles

**Fig. 2:**
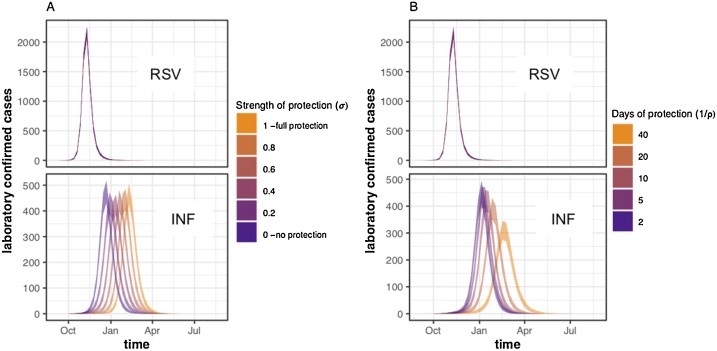
Mean weekly incidence of observed cases in under 5 s (sum of age groups 0-1 and 2-4) from simulations with A) varying σ values and a fixed protection duration of 10 days (ρ = 0.1), and B) varying ρ values, and a fixed σ of 0.5. Simulations were run and sampled 1000 times for each parameter set and the shaded windows are the 95 % quantiles for each week. In both A and B the top panel shows the observed cases for RSV, and the lower panel the cases for Influenza.

Altering the strength or duration of cross-protection did not notably affect the timing or shape of the RSV epidemic (Fig. 2), due to the higher transmission rate and earlier start of RSV in our scenarios. However, increasing the strength or duration of interaction delayed the influenza peak. The total number of influenza infections in the youngest two age groups did not change (percentage difference <1% between σ = 1 and σ = 0) with the strength of interaction (Supplement Section 8). Increasing the duration of cross-protection resulted in an 11 % lower total number of infections from the shortest (2 days) to longest (40 days) duration of cross-protection (Supplement Section 8). Plots showing the epidemic curves for each infectious compartment are shown in in Supplement Section 9.

### 3.2 Correlation analysis

**Fig. 3:**
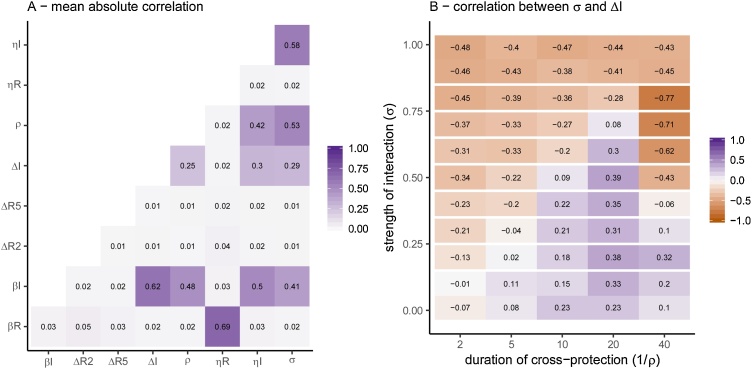
A) Mean Pearson correlation coefficient between parameters. B) Correlation coefficient between σ ( strength of cross-protection) and $\Delta_{INF}$ (start day of influenza). This is shown for 1 simulation, but the patterns were similar for all (Supplementary Section 12).

The most strongly correlated parameters were consistently the transmission rate for RSV $\left(\beta_{RSV}\right)$ with the RSV season start time ($\eta_{RSV}$) and the transmission rate for influenza ($\beta_{INF}$) with the detection rate for influenza (${\text{Δ}}_{INF}$) and the start of the influenza season ($\eta_{INF}$) (Fig. 3A). The correlation between parameters changed dependent on the values of the interaction parameters, an example of which is shown in Fig. 3B, where the correlation coefficient between the strength of interaction (σ) and the proportion of influenza cases detected (${\text{Δ}}_{INF}$) varies depending on the values of σ and ρ. As the strength of interaction decreases (as σ→0), the correlation between the strength of interaction and the proportion of influenza cases detected becomes more positive. The correlation changes across the interaction parameters for other parameter combinations are shown in in the Supplement Section 10, and matrices for individual simulations are in supplement section 12.

### 3.3 Inferring the strength of cross-protection (σ)

**Fig. 4:**
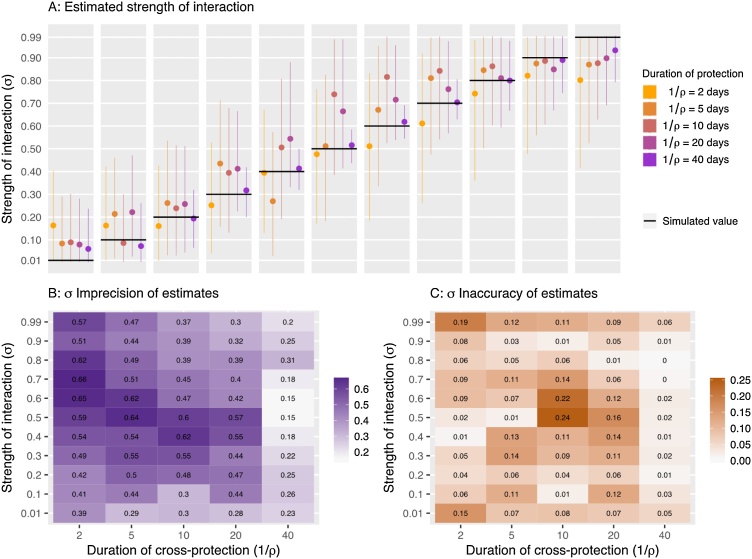
A) Estimated σ values for simulations with different σ and ρ values. Median value and 95 % CI are shown. The black line is the simulated (true) value of σ in each case. B) Imprecision of σ estimates calculated as the 95 % quantile range. C) Inaccuracy of the σ estimates, calculated as the difference between the posterior median and the true value.

Across simulations, the imprecision and inaccuracy of the estimated strength of cross-protection (σ) varied (Fig. 4), with the imprecision ranging from 0.15 to 0.66 (where 1 is poor precision) and average imprecision decreasing as the duration of protection (ρ) increased. We did not observe a trend in the inaccuracy of the parameter estimates and they ranged from 0 to 0.24. However, the lowest value tested (σ = 0.01) was overestimated in each simulation and the highest value tested (σ = 0.99) was underestimated, showing that the extreme values are less well estimated. This may be due to the true value being very close to the limit.

### 3.4 Inferring the duration of cross-protection (1/ρ)

**Fig. 5:**
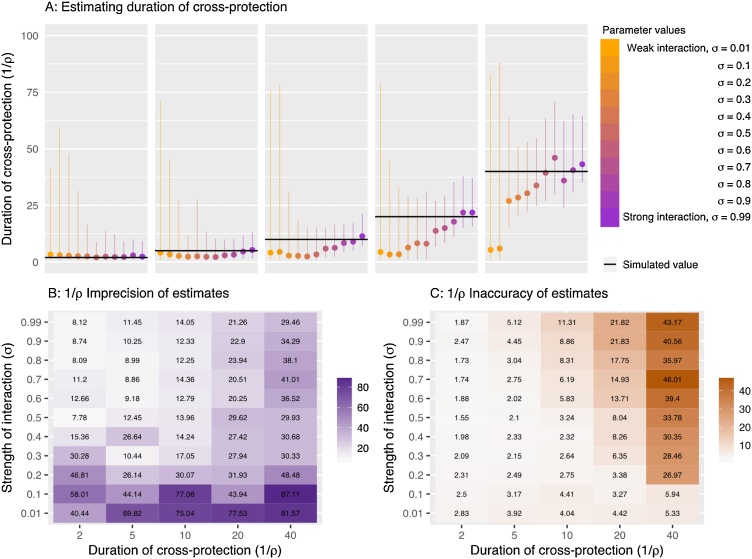
A) Estimated 1/ ρ values for simulations with different σ and ρ. Lines represent 95 % quantiles of the posterior sample and the circle represents the median value. The black line shows the true 1/ ρ value in each case. B) Imprecision of ρ estimates calculated as the 95 % quantile range. C) Inaccuracy of the ρ estimates, calculated as the difference between the posterior median and the true value.

The imprecision of the estimated duration of cross protection ranged from 7 to 87 days (Fig. 5). Estimates were generally less precise when the period of cross-protection is longer (Fig. 5). In 70 % of our simulations where  σ interaction reduced the transmission rate by no more than 10 % (i.e. σ = 0.1 or 0.01) the duration of protection estimates exceeded an imprecision of 50 days. For scenarios assuming stronger competition estimates were much more precise. Indeed, one would expect that once the strength of competition is negligibly small the duration of such protection would be largely irrelevant. ρ estimates increased for simulations generated with longer duration of protection (smaller *ρ*).

### 3.5 Variation between replicates

**Fig. 6:**
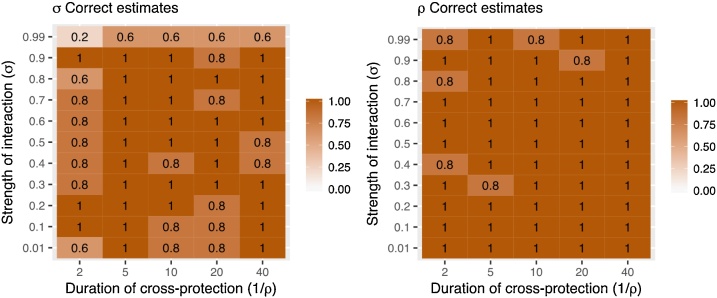
Proportion of simulations where the true value of σ (A) and ρ (B) was included in the 95 % CI of the posterior estimate.

Each parameter set was used to generate a further four replicate simulations of the observation process. Of these 275 simulations (5 replicates of 55 parameter combinations), 259 reached convergence at the cut-off point (see Supplement Section 11). The true parameter values were included in the 95 % CI in the majority of replicates (Fig. 6). The true value of ρ was not included in the 95 % CI in 6 simulations (2%), whereas the true value of σ was not included in the 95 % CI in 31 simulations (11 %). These simulations were more concentrated in areas with extreme interaction strengths (0.99 and 0.01) and very short duration of protection. We conclude from this that the stochastic variation in the simulation of the observation can occasionally result in difficulty estimating the true value of the parameter.

## 4 Discussion

We tested whether a transmission model including competitive interaction between RSV and influenza is identifiable from a single season of simulated high-quality surveillance data. We determined that it is possible to re-estimate strength and duration of interaction in most tested scenarios, although often imprecise due to large credible intervals, but that there are some areas of parameter space where posterior estimates are potentially misleading, particularly when the strength of interaction is assumed to be low or the duration of interaction short. However, we only estimated the parameters from information from one season at a time, and without strong priors.

While we are the first to test robustness of RSV and influenza competition inference, other identifiability studies, e.g. on Rift Valley Fever, have previously highlighted the importance of robustness testing to avoid misleading conclusions stemming largely from insufficient power of the data to inform the model parameters of interest (Kao and Eisenberg, 2018; Tuncer et al., 2016). Structural and practical identifiability analysis have also been used to select appropriate models, given the data available (Tuncer et al., 2018; Roosa and Chowell, 2019), for example a study that evaluated six different Zika models, and the identifiability of parameters within each (Tuncer et al., 2018).

Our analysis shows that there are potentially misleading results at extreme competition values, and it is almost impossible to get a “null estimate” for the strength of competition from this study. Evidence from mouse models suggest that the duration of RSV cross protection following influenza infection may last more than two weeks (Walzl et al., 2000; Hamilton, 2021) which, under the assumption that the duration of cross-protection is non-differential to the initiating virus, may suggest that the imprecision of our estimates at short durations of cross protection is unlikely to be a key risk for inference. However it may not be possible to distinguish such competition from no competition in our model, if the strength of the competition is low. We deliberately used uninformative priors for this parameter in order to be able to fully explore its identifiability, however, subsequent work may further improve precision of estimates by including prior estimates based on published evidence. This may also reduce the correlation of estimated parameters, which has challenged convergence in our simulations. Similarly, mouse models have suggested strong modulation of the RSV immune response if preceded by an influenza infection (Walzl et al., 2000), which may suggest that difficulties in our inference in scenarios that assume very small amounts of competition may not be the most relevant.

For parameter combinations where the simulated parameter value could not be re-estimated, we found that despite the relatively high assumed sample size stochastic noise from the observation model can occasionally result in incorrect estimates. This implies that inference based on a single season may be misleading purely because of the observational process associated with surveillance, however, including multiple seasons of observation should limit problems stemming from the observation process alone and further increase accuracy of estimates.

We assumed that the RSV–influenza interaction was bidirectional; particularly we assume that the strength and duration of interaction that influenza exhibits on RSV is the same as vice versa. Given that the proposed mechanisms for interaction are not virus specific this seems reasonable, and is supported by studies looking at the shift in RSV epidemics following the early 2009 influenza pandemic (Mak et al., 2012; Hirsh et al., 2014; Gröndahl et al., 2014; Casalegno et al., 2010). However, the RSV epidemic in the UK typically precedes influenza and similarly we only investigate such scenario. Therefore, in this work we can only estimate the competition of RSV on influenza dynamics and do not have power to estimate the other direction. Hence our results are applicable for considerations around RSV vaccine introduction but should be treated cautiously for any studies interested in the impact of Influenza on the transmission dynamics of RSV.

This model did not include multiple strains of either RSV or influenza, which could have an impact on the interaction dynamics, as the interaction may differ between strains. Including strains would significantly increase the complexity of the model (see review on strain interaction models (Kucharski and Gog, 2012)), which we think would have rendered it unidentifiable. In addition, the aim was to assess the practical identifiability of the model parameters that govern viral interaction from routine surveillance data, and in many scenarios the surveillance data does not record strain type. The biological mechanism underpinning the period of cross-immunity is that of viral infection-induced protection, which is potentially induced by many viruses so may not be specific to RSV and influenza, and may not differ between influenza subtypes/strains.

We ran the model for one season at a time in order to reduce the complexity, as in other influenza models (Baguelin et al., 2013). We captured influenza immunity from previous seasons in the proportion of individuals susceptible for influenza at the start of the season, and RSV immunity is considered to last less than a year (Weber et al., 2001), so we simplified to a single season but included the major multi-season effects. A further sensitivity analysis could be to vary the susceptibility of individuals to influenza at the start of the year, in order to simulate different dominant influenza strains. We have however not included this, as our aim here was to look at the identifiability of parameters, and these differences would be taken into account when fitting to surveillance data from different seasons. Ideally, we would fit to multiple seasons of surveillance data, in order to account for variations by year. In the model we assumed a constant, age-dependent observation rate, as in other influenza models (Magal and Webb, 2018). Time varying reporting rates would substantially hinger inference, in fact a previous study comparing model fit of age-dependent vs time and age-dependent reporting rates concluded it was not possible to prefer one model terms of fit alone (Dorigatti et al., 2012), so we assume age-dependent only reporting rates for simplicity. We did not include additional seasonal effects in the model. While no or small effects have been reported for RSV and seasonal factors (Tian et al., 2017; Hogan et al., 2016), there is stronger evidence for the impact of climatic factors on influenza transmission, particularly ambient temperature and absolute humidity (Shaman et al., 2010; Shaman and Kohn, 2009; Lowen and Steel, 2014). While this does not affect our results on identifying parameters from simulated data, it should be noted as a potential confounder when estimating these parameters using surveillance data.

Further data may help to identify parameters in the model where it currently has difficulties. Data on the frequency of co-infections would allow us to use stronger priors for the strength of interaction, as well as providing an informative data source to fit the model to. Further information on the circulation of RSV, as opposed to only clinical cases, is also important due to the current uncertainty in infection numbers. In addition, surveillance systems would ideally provide daily data on RSV and influenza cases, giving us more granularity and potentially allowing us to identify all areas of parameter space.

Behavioural changes may also impact respiratory viral circulation, after infection with a virus (staying inside while recovering), or large-scale behavioural change due to restrictions (social distancing measures in response to the SARS-CoV-2 pandemic). Whilst we do not investigate these mechanisms in this paper, such changes can have drastic impacts, such as the largely absent 2020 influenza season in Australia (Sullivan et al., 2020).

Overall, this study shows that in principle interaction parameters can be estimated from high quality surveillance-like data using mathematical models, although the precision and accuracy of the estimates varies depending on the scenario and stochasticity in the surveillance data. More power to reliably infer parameters may be available if fitting multiple seasons. It also highlights the importance of validating complex models, especially in light of the rapid development of models in emergency situations, which can have large impacts on public policy (Panovska-Griffiths, 2020).

## Author summary

Influenza and Respiratory Syncytial Virus (RSV) cause a large disease burden. Rather than acting independently these viruses may interact, meaning that infection with one decreases the likelihood of infection with the other. While this could have important implications for control strategies, the evidence for the strength of the interaction and its importance for public health is largely based on ecological studies, and it is not clear that surveillance data are sufficient to determine if interaction exists, and if so, how long the effect last. To test this assumption we used a mathematical model to simulate RSV and Influenza surveillance data and back-infer the strength and duration of interaction used to generate the data. We found that in the majority of cases it was possible to determine the strength and duration of interaction from even a single season of high-quality surveillance. However, we also showed that for extreme parameter values, model estimates may be unreliable despite a seemingly good fit to the data and hence highlight the importance of *a priori* model validation for similar analyses.

## Author Statement

**Rosalind M Eggo:** Conceptualisation, Methodology, Writing – Review and Editing, Supervision, Visualization.

**Stefan Flashe**: Conceptualisation, Methodology, Writing – Review and Editing, Supervision, Visualization.

**Amanda Minter:** Validation, Methodology, Writing – Review and Editing.

**Naomi R Waterlow:** Conceptualisation, Methodology, Software, Validation, Formal Analysis, Writing – Original Draft, Visualization.

## Declaration of Competing Interest

The authors report no declarations of interest.

## References

[bib0005] Ascough S., Paterson S., Chiu C. (2018). Induction and subversion of human protective immunity: contrasting influenza and respiratory syncytial virus. Front. Immunol..

[bib0010] Baguelin M., Jit M., Miller E., Edmunds W.J. (2012). Health and economic impact of the seasonal influenza vaccination programme in England. Vaccine.

[bib0015] Baguelin M. (2013). Assessing optimal target populations for influenza vaccination programmes: an evidence synthesis and modelling study. PLoS Med..

[bib0020] Bloom-Feshbach K. (2013). Latitudinal variations in seasonal activity of influenza and respiratory syncytial virus (RSV): a global comparative review. PLoS One.

[bib0025] Casalegno J.S. (2010). Impact of the 2009 influenza a(H1N1) pandemic wave on the pattern of hibernal respiratory virus epidemics, France, 2009. Eurosurveillance.

[bib0030] Cauchemez S., Carrat F., Viboud C., Valleron A.J., Boëlle P.Y. (2004). A Bayesian MCMC approach to study transmission of influenza: application to household longitudinal data. Stat. Med..

[bib0035] Chowell G. (2011). Characterizing the epidemiology of the 2009 influenza A/H1N1 pandemic in Mexico. PLoS Med..

[bib0040] Cori A. (2012). Estimating influenza latency and infectious period durations using viral excretion data. Epidemics.

[bib0045] Dorigatti I., Cauchemez S., Pugliese A., Ferguson N.M. (2012). A new approach to characterising infectious disease transmission dynamics from sentinel surveillance: application to the Italian 2009-2010 A/H1N1 influenza pandemic. Epidemics.

[bib0050] Eddelbuettel D., Francois R. (2011). Rcpp: seamless R. And C++ integration. J. Stat. Softw..

[bib0055] Ferguson N.M., Galvani A.P., Bush R.M. (2003). Ecological and immunological determinants of influenza evolution. Nature.

[bib0060] Ferguson N.M. (2005). Strategies for containing an emerging influenza pandemic in Southeast Asia. Nature.

[bib0065] Funk S. (2018). socialmixr: Social Mixing Matrices for Infectious Disease Modelling.

[bib0070] Gröndahl B. (2014). The 2009 pandemic influenza A(H1N1) coincides with changes in the epidemiology of other viral pathogens causing acute respiratory tract infections in children. Infection.

[bib0075] Hall C.B., Douglas R.G., Geiman J.M. (1976). Respiratory syncytial virus infections in infants: quantitation and duration of shedding. J. Pediatr..

[bib0080] Hamilton, J. Club cells surviving influenza A virus infection induce temporary nonspecific antiviral immunity. 10.1073/pnas.1522376113 PMC4833272 27001854

[bib0085] Hayden F.G. (1999). Use of the oral neuraminidase inhibitor oseltamivir in experimental human influenza. JAMA.

[bib0090] Henderson F.W., Collier A.M., Clyde W.A., Denny F.W. (1979). Respiratory-syncytial-virus infections, reinfections and immunity. N. Engl. J. Med..

[bib0095] Hirsh S. (2014). Epidemiological changes of respiratory syncytial virus (RSV) infections in Israel. PLoS One.

[bib0100] Hogan A.B. (2016). Time series analysis of RSV and bronchiolitis seasonality in temperate and tropical Western Australia. Epidemics.

[bib0105] Kao Y.-H., Eisenberg M.C. (2018). Practical unidentifiability of a simple vector-borne disease model: implications for parameter estimation and intervention assessment. Epidemics.

[bib0110] Kelly H., Barry S., Laurie K., Mercer G. (2010). Seasonal influenza vaccination and the risk of infection with pandemic influenza: a possible illustration of nonspecific temporary immunity following infection. Eurosurveillance.

[bib0115] Kucharski A.J., Gog J.R. (2012). Age profile of immunity to influenza: effect of original antigenic sin. Theor. Popul. Biol..

[bib0120] Kucharski A.J., Andreasen V., Gog J.R. (2016). Capturing the dynamics of pathogens with many strains. J. Math. Biol..

[bib0125] Lafond K.E. (2016). Global role and burden of influenza in pediatric respiratory hospitalizations, 1982–2012: a systematic analysis. PLoS Med..

[bib0130] Laurie K.L. (2015). Interval between infections and viral hierarchy are determinants of viral interference following influenza virus infection in a ferret model. J. Infect. Dis..

[bib0135] Lee Y.J. (2018). Non-specific effect of vaccines: immediate protection against respiratory syncytial virus infection by a live attenuated influenza vaccine. Front. Microbiol..

[bib0140] Lowen A.C., Steel J. (2014). Roles of humidity and temperature in shaping influenza seasonality. J. Virol..

[bib0145] Magal P., Webb G. (2018). The parameter identification problem for SIR epidemic models: identifying unreported cases. J. Math. Biol..

[bib0150] Mak G.C., Wong A.H., Ho W.Y.Y., Lim W. (2012). The impact of pandemic influenza A (H1N1) 2009 on the circulation of respiratory viruses 2009-2011. Influenza Other Respi. Viruses.

[bib0155] Meningher T. (2014). Relationships between A(H1N1)pdm09 influenza infection and infections with other respiratory viruses. Influenza Other Respi. Viruses.

[bib0160] Moore H.C., Jacoby P., Hogan A.B., Blyth C.C., Mercer G.N. (2014). Modelling the seasonal epidemics of respiratory syncytial virus in young children. PLoS One.

[bib0165] Mossong J. (2008). Social contacts and mixing patterns relevant to the spread of infectious diseases. PLoS Med..

[bib0170] Nokes D.J. (2004). Respiratory syncytial virus epidemiology in a birth cohort from Kilifi District, Kenya: infection during the first year of life. J. Infect. Dis..

[bib0175] Novavax Announces Topline Results from Phase 3 PrepareTM Trial of ResVax™ for Prevention of RSV Disease in Infants via Maternal Immunization Novavax Inc. - IR Site. Available at: https://ir.novavax.com/news-releases/news-release-details/novavax-announces-topline-results-phase-3-preparetm-trial. (Accessed: 29th August 2019).

[bib0180] Ohuma E.O. (2012). The natural history of respiratory syncytial virus in a birth cohort: the influence of age and previous infection on reinfection and disease. Am. J. Epidemiol..

[bib0185] Opatowski L., Baguelin M., Eggo R.M. (2017). Review Influenza Interaction with Cocirculating Pathogens, and its Impact on Surveillance.

[bib0190] Panovska-Griffiths J. (2020). Can mathematical modelling solve the current Covid-19 crisis?. BMC Public Health.

[bib0195] Pascalis H. (2012). Intense Co-circulation of non-influenza respiratory viruses during the first wave of pandemic influenza pH1N1/2009: a cohort study in Reunion Island. PLoS One.

[bib0200] Pinky L., Dobrovolny H.M. (2016). Coinfections of the respiratory tract: viral competition for resources. PLoS One.

[bib0205] Pneumonia Etiology Research for Child Health (PERCH) Study Group, K. L (2019). Causes of severe pneumonia requiring hospital admission in children without HIV infection from Africa and Asia: the PERCH multi-country case-control study. Lancet (London, England).

[bib0210] R Core Team (2018). R: A Language and Environment for Statistical Computing.

[bib0215] Reeves R.M. (2017). Estimating the burden of respiratory syncytial virus (RSV) on respiratory hospital admissions in children less than five years of age in England, 2007-2012. Influenza Other Respi. Viruses.

[bib0220] Respiratory infections: laboratory reports 2019 - GOV.UK. Available at: https://www.gov.uk/government/publications/respiratory-infections-laboratory-reports-2019. (Accessed: 11th January 2021).

[bib0225] Roosa K., Chowell G. (2019). Assessing parameter identifiability in compartmental dynamic models using a computational approach: application to infectious disease transmission models. Theor. Biol. Med. Model..

[bib0230] Shaman J., Kohn M. (2009). Absolute humidity modulates influenza survival, transmission, and seasonality. Proc. Natl. Acad. Sci. U. S. A..

[bib0235] Shaman J., Pitzer V.E., Viboud C., Grenfell B.T., Lipsitch M. (2010). Absolute humidity and the seasonal onset of influenza in the Continental United States. PLoS Biol..

[bib0240] Shi T. (2017). Global, regional, and national disease burden estimates of acute lower respiratory infections due to respiratory syncytial virus in young children in 2015: a systematic review and modelling study. Lancet (London, England).

[bib0245] Shinjoh M., Omoe K., Saito N., Matsuo N., Nerome K. (2000). In vitro growth profiles of respiratory syncytial virus in the presence of influenza virus. Acta Virol..

[bib0250] Soetaert K., Petzoldt T., Woodrow S. (2010). Solving differential equations in r: package deSolve. J. Stat. Softw..

[bib0255] Sullivan S.G. (2020). Where has all the influenza gone? The impact of COVID-19 on the circulation of influenza and other respiratory viruses, Australia, March to September 2020. Eurosurveillance.

[bib0260] Tian D.D., Jiang R., Chen X.J., Ye Q. (2017). Meteorological factors on the incidence of MP and RSV pneumonia in children. PLoS One.

[bib0265] Tuncer N., Gulbudak H., Cannataro V.L., Martcheva M. (2016). Structural and Practical Identifiability Issues of Immuno-Epidemiological Vector–Host Models with Application to Rift Valley Fever. Bull. Math. Biol..

[bib0270] Tuncer N., Marctheva M., LaBarre B., Payoute S. (2018). Structural and practical identifiability analysis of Zika epidemiological models. Bull. Math. Biol..

[bib0275] van Asten L. (2016). Early occurrence of influenza A epidemics coincided with changes in occurrence of other respiratory virus infections. Influenza Other Respi. Viruses.

[bib0280] Velasco-Hernández J.X., Núñez-López M., Comas-García A., Cherpitel D.E.N., Ocampo M.C. (2015). Superinfection between influenza and RSV alternating patterns in San Luis Potosí State, México. PLoS One.

[bib0285] Walzl G., Tafuro S., Moss P., Openshaw P.J., Hussell T. (2000). Influenza virus lung infection protects from respiratory syncytial virus-induced immunopathology. J. Exp. Med..

[bib0290] Weber A., Weber M., Milligan P. (2001). Modeling epidemics caused by respiratory syncytial virus (RSV). Math. Biosci..

[bib0295] Weekly national flu reports: 2018 to 2019 season - GOV.UK. Available at: https://www.gov.uk/government/statistics/weekly-national-flu-reports-2018-to-2019-season. (Accessed: 11th January 2021).

[bib0300] Yang Y. (2012). Influenza A/H1N1 2009 Pandemic and Respiratory Virus Infections, Beijing, 2009-2010. PLoS One.

[bib0305] Zhao H. (2014). A new laboratory-based surveillance system (Respiratory Datamart System) for influenza and other respiratory viruses in England: results and experience from 2009 to 2012. Eurosurveillance.

