# Supplementary material for: Competition between RSV and influenza: Limits of modelling inference from surveillance data
Source: Epidemics. 2021 Jun;35:100460. doi: 10.1016/j.epidem.2021.100460 (PMC8193815; doi:10.1016/j.epidem.2021.100460)
Supplement: Supplementary file 1 [file mmc1.docx]

Supplementary information for

**“Interactions between RSV and influenza: limits of modelling inference from surveillance data “**

*Naomi R Waterlow^1^, Stefan Flasche^1^, Amanda Minter^1^, Rosalind M Eggo^1^*

*^1^Department of Infectious Disease Epidemiology, London School of Hygiene and Tropical Medicine, UK*

Table of Contents:

1. Model equations
2. Full model diagram
3. R0 calculation
4. Susceptibility to RSV
5. Calibrating detection rates
6. Sensitivity of introductions
7. Priors and parameter limits
8. Simulated data total case number
9. Individuals within infectious compartments
10. Parameter correlations
11. Convergence
12. Individual simulation analysis
13. Varying the R0
14. *Model equations*

$$\begin{aligned} \lambda_{{RSV}_{ij}}={\sum\beta_{RSV}\alpha}_{ij}I_{{RSV}_{j}} \end{aligned}(1)$$

$$\begin{aligned} \lambda_{{INF}_{ij}}={\sum\beta_{INF}\alpha}_{ij}I_{{INF}_{j}} \end{aligned}(2)$$

$$\frac{dSS_{i}}{dt}={- \tau_{i}\lambda}_{{RSV}_{i}}SS_{i} {- \lambda}_{{INF}_{i}}{SS}_{i}$$

$$\frac{d{IS}_{i}}{dt}= {\tau_{i}\lambda}_{{RSV}_{i}}{SS}_{i} {- (1-\sigma)\lambda}_{{INF}_{i}}{IS}_{i}-\gamma_{RSV}{IS}_{i}$$

$$\frac{d{PS}_{i}}{dt}= \gamma_{RSV}{IS}_{i}-\rho PS_{i}-(1-\sigma){\lambda_{\mathrm{INF}}}_{i}PS_{i}$$

$$\frac{d{RS}_{i}}{dt}= \rho PS_{i}-{\lambda_{\mathrm{INF}}}_{i}RS_{i}$$

$$\frac{d{SI}_{i}}{dt}= \lambda_{{INF}_{i}}{SS}_{i}- {{(1-\sigma)\tau}_{i}\lambda}_{{RSV}_{i}}{SI}_{i}-\gamma_{INF}{SI}_{i}$$

$$\frac{d{II}_{i}}{dt}= {(1-\sigma)\lambda}_{{INF}_{i}}{IS}_{i}+(1-{\sigma)\tau_{i}\lambda}_{{RSV}_{i}}{SI}_{i}-\gamma_{INF}{II}_{i}- \gamma_{RSV}{II}_{i}$$

$$\frac{d{PI}_{i}}{dt}= \lambda{PS}_{i}-\gamma_{INF}{PI}_{i}+\gamma_{RSV}{II}_{i}+\lambda_{{INF}_{i}}{RS}_{i}$$

$$\frac{d{SP}_{i}}{dt}= \gamma_{INF}{SI}_{i}-\rho{SP}_{i}-(1-\sigma){{\tau_{i}\lambda}_{\mathrm{RSV}}}_{i}{SP}_{i}$$

$$\frac{d{IP}_{i}}{dt}= {(1-\sigma)\tau_{i}\lambda}_{RSV}{SP}_{i}-\gamma_{RSV}{IP}_{i}+\gamma_{INF}{II}_{i}+{\tau_{i}\lambda}_{{RSV}_{i}}{SR}_{i}$$

$$\frac{d{SR}_{i}}{dt}= \rho{SP}_{i}-{{\tau_{i}\lambda}_{\mathrm{RSV}}}_{i}{SR}_{i}$$

$$\frac{d{RR}_{i}}{dt}= \gamma_{RSV}{IP}_{i}+\gamma_{INF}{PI}_{i}$$

States

The first letter of the state indicates the state for RSV, the second letter indicates the state for Influenza.

E.g. $SS_{i}$is shorthand for $S_{RSV,i}S_{INF,i}$

S - Susceptible

I - Infected

P – Partially cross protected

R – Recovered

Subscripts

$INF$ - Influenza

$R$SV – Respiratory Syncytial Virus

Parameters

$\lambda_{i,j}$ – transmission rate between age groups I and J

$\beta$ – transmission rate

$\alpha_{ij}$ – contact rate between group I and j

$\tau_{i}$ - age group susceptibility to RSV

$\sigma$ – level of cross-protection

$\gamma$ – rate of recovery

$\rho$ – rate of loss of cross-protection

1. *Full model Diagram*

**

Figure S 1: Model diagram for RSV and Influenza (INF) demonstrating all age groups. Population can be Susceptible (S), Infected, (I), Protected (P) or Recovered (R) to each virus. For each virus, following infection, infectiousness wanes at a constant rate, and the population enters the P state. Here they are immune to the virus they were infected by and protected to a varying extent against infection from the second virus. This protection wanes at a rate which we change, and the population enters the R compartment. In the R compartment the population is immune to the virus it was infected by, but not the other virus. Parameters are: recovery rate for RSV and Influenza ($\gamma_{RSV}$and $\gamma_{INF}$), age susceptibility to infection ($\tau_{i}$), the transmission parameters for RSV and Influenza ($\beta_{RSV,i}$ and $\beta_{INF,i}$), strength of cross-protection ($\sigma$) and rate of protection loss ($\rho$). Values are given in Table 1. The age is denoted by the subscript (i).

1. *R_0_ calculation*

The R_0_ for each virus was calculated using the method described in Diekmann *et al (2009)*^1^, assuming no interaction between the viruses. The R_0_ is the dominant eigenvalue of the matrix

$$-T\Sigma^{-1}$$

where $T$ is the transmission part of the Jacobean matrix, describing the production of new infections, and $\Sigma$ is the transition part, describing changes in state^1^. See reference for further details.

$$T_{INF}= \left[ \begin{matrix} \beta_{INF}*\alpha_{i,j} & \cdots& \beta_{INF}*\alpha_{i,j} \\ \vdots& \ddots& \vdots\\ \beta_{INF}*\alpha_{i,j} & \cdots& \beta_{INF}*\alpha_{i,j} \end{matrix} \right] T_{RSV}= \left[ \begin{matrix} {\tau_{j}*\beta}_{RSV}*\alpha_{i,j} & \cdots& {\tau_{i}*\beta}_{RSV}*\alpha_{i,j} \\ \vdots& \ddots& \vdots\\ \tau_{j}*\beta_{RSV}*\alpha_{i,j} & \cdots& {\tau_{j}*\beta}_{RSV}*\alpha_{i,j} \end{matrix} \right]$$

$$\Sigma_{INF}= \left| \begin{matrix} \gamma_{INF} & 0 & 0 & 0 & 0 \\ 0 & \gamma_{INF} & 0 & 0 & 0 \\ 0 & 0 & \gamma_{INF} & 0 & 0 \\ 0 & 0 & 0 & \gamma_{INF} & 0 \\ 0 & 0 & 0 & 0 & \gamma_{INF} \end{matrix} \right| \Sigma_{\mathrm{RSV}}= \left| \begin{matrix} \gamma_{RSV} & 0 & 0 & 0 & 0 \\ 0 & \gamma_{RSV} & 0 & 0 & 0 \\ 0 & 0 & \gamma_{RSV} & 0 & 0 \\ 0 & 0 & 0 & \gamma_{RSV} & 0 \\ 0 & 0 & 0 & 0 & \gamma_{RSV} \end{matrix} \right|$$

Subscripts

$INF$ - Influenza

$RSV$ – Respiratory Syncytial Virus

Parameters

$\beta$–transmission rate

$\alpha_{ij}$ – contact rate between group I and j

$\tau_{i}$ - age group susceptibility to RSV

$\gamma$ – rate of recovery

1. *Susceptibility to RSV*

RSV susceptibility differs with age, as indicated by a longitudinal study by Henderson et al. (1979) in which they determined that at 1st exposure 98.4% of children became infected, at second exposure 74.5% of children became infected and at 3rd exposure 65.4% of children became infected^2^. As it is estimated that almost all children are infected by 24 months of age^3^ , we used these figures as the reduced susceptibility of age groups (ages 0-1 = 100% susceptible, ages 2-4 = 75% susceptible, ages 5 and over = 65% susceptible).

1. *Calibrating detection rates*

We calibrated the detection and transmission rates for the viruses in the under-five population to observed values in the UK, by matching simulation output to epidemic peak incidence and duration^4^. In addition, we set the rates for RSV to different values in each age group, as younger infants have been shown to be more likely to present with severe symptoms^5^ .

| **Parameter** | **Symbol** | **Value** |
| --- | --- | --- |
| Proportion of RSV infections in ages 0-1 hospitalised | $\Delta_{R1}$ | 0.004 |
| Proportion of RSV infections in ages 2-4 hospitalised | $\Delta_{R2}$ | 0.001 |
| Proportion of Influenza infections in ages 0-4 hospitalised | $\Delta_{I}$ | 0.002 |

Table S 1: parameters used in the model for the proportion of infections detected by age group and virus.

1. *Sensitivity of introductions*

Introduction of influenza as a one of infection at season start time ($\eta_{I}$) was also tested to look at the sensitivity of the assumption. With a single introduction the influenza epidemic was supressed for the whole season at certain parameter values. This occurred when there was strong interaction between the two viruses and the introduction of influenza occurred during the peak of the RSV epidemic. As such behaviour is not seen in England, we judged the assumption of low-level constant introduction to be more valid.

Parameter limits were kept constant, except for the limit on $\sigma$, which either ranged from 0 to 1 (competitive interaction only) or from 1 to -1 (allowing for synergistic as well as competitive interaction). Although we do not think a synergistic relationship between influenza and RSV is likely, we thought including it may allow the chain to more effectively explore the parameter space. However, for values between 0.2 and 0 no MCMC chains converged when allowing for synergistic interaction, so in all subsequent runs $\sigma$ was limited between 0 and 1.

We ran a sensitivity analysis on the start times of the viruses, running / fitting extra simulations with influenza and RSV starting on the same day and 20 days apart. These simulations were run as in the main paper, however limited to 250000 iterations after burn in. When the viruses were seeded on the same day (and there was greater overlap of epidemics), the estimates for both interaction parameters were more precise (Figure S 2, Figure S 3) than the standard simulations. Conversely, when influenza was seeded later than standard, the estimates were less precise.

Figure S 2: Estimated ρ values from MCMC inference for simulations with different σ and ρ values. Median value and 95% CI are shown. The black line is the simulated (true) value of ρ in each case. The different plots show simulations with different intervals between influenza and RSV start times.

Figure S 3:Estimated σ values from MCMC inference for simulations with different σ and ρ values. Median value and 95% CI are shown. The black line is the simulated (true) value of σ in each case. The different plots show simulations with different intervals between influenza and RSV start times.

1. *Priors and parameter limits*

| **Parameter** | **Symbol** | **Prior distribution** | **Lower Limit** | **Upper Limit** |
| --- | --- | --- | --- | --- |
| *R_0_* for RSV  used to calculate basic transmission rate | $\beta_{0,RSV}$ | Normal distribution, mean=3, sd=0.6 | 0 | Infinity |
| *R_0_* for Influenza  used to calculate basic transmission rate | $\beta_{0,INF}$ | Lognormal distribution, mean=0.3, sd=0.6, shifted by 1 | 0 | Infinity |
| Time of first RSV infection | $\eta_{RSV}$ | Uniform between day 0 and day 60 | 0 | 60 |
| Time of first influenza infection | $\eta_{INF}$ | Uniform between day 0 and day 60 | 0 | 60 |
| Proportion of RSV infections in ages 0-1 hospitalised | $\Delta_{RSV,1}$ | Uniform | 0 | Infinity |
| Proportion of RSV infections in ages 2-4 hospitalised | $\Delta_{RSV,2}$ | Uniform | 0 | Infinity |
| Proportion of Influenza infections in ages 0-4 hospitalised | $\Delta_{INF}$ | Uniform | 0 | Infinity |
| Strength of interaction | $\sigma$ | Uniform between 0 and 1 | 0 | 1 (or -1 for sensitivity check – see supplementary section 6) |
| Rate of loss of cross-protection | $\rho$ | Uniform between 0.01 and 1 | 0 | 1 |

Table S 2: Prior distributions and limits for the parameters used in the model during the Markov Chain Monte Carlo (MCMC) fitting process. sd = standard deviation.

1. *Simulated data total case numbers*

| 1/duration of cross-protection ($\rho$) | Number of RSV cases | Number of Influenza cases |
| --- | --- | --- |
| 2 | 3182023 | 1294980 |
| 5 | 3181955 | 1292596 |
| 10 | 3182035 | 1283034 |
| 20 | 3182200 | 1245207 |
| 40 | 3182284 | 1152700 |

Table S 3: shows the total number of infections with different duration of cross-protection. The level of cross-protection is fixed at $\sigma$ = 0.5.

| Strength of cross-protection ($\sigma$) | Number of RSV cases | Number of Influenza cases |
| --- | --- | --- |
| 1 | 3182422 | 1303752 |
| 0.8 | 3182341 | 1297009 |
| 0.6 | 3182152 | 1287792 |
| 0.4 | 3181936 | 1279325 |
| 0.2 | 3182025 | 1281968 |
| 0 | 3183181 | 1314065 |

Table S 4: shows the total number of infections with different duration of cross-protection. The duration of cross-protection is fixed at $1/\rho$ = 10.

1. *Individuals within infectious compartments*

**

Figure S 4: Individuals within each infectious compartment for the age group 0-1. Horizontal facets show the ρ value, colours the σ value. Vertical facets show the different infectious compartments.

Figure S 5: Individuals within each infectious compartment for the age group 2-4. See Figure S2 legend for more details.

Figure S 6: Individuals within each infectious compartment for the age group 5-15. See Figure S2 legend for more details.

**

Figure S 7: Individuals within each infectious compartment for the age group 16-64. See Figure S2 legend for more details.

Figure S 8: Individuals within each infectious compartment for the age group 65+. See Figure S2 legend for more details.

1. *Parameter correlations*

**

Figure S 9:Pearson correlation coefficients for parameter combinations specified in the title, with changing interaction parameters (ρ and σ).

**

Figure S 10: Pearson correlation coefficients for parameter combinations specified in the title, with changing interaction parameters (ρ and σ).

**

Figure S 11: Pearson correlation coefficients for parameter combinations specified in the title, with changing interaction parameters (ρ and σ).

**

Figure S 12: Pearson correlation coefficients for parameter combinations specified in the title, with changing interaction parameters (ρ and σ).

**

Figure S 13: Pearson correlation coefficients for parameter combinations specified in the title, with changing interaction parameters (ρ and σ).

**

Figure S 14: Pearson correlation coefficients for parameter combinations specified in the title, with changing interaction parameters (ρ and σ).

**

Figure S 15: Pearson correlation coefficients for parameter combinations specified in the title, with changing interaction parameters (ρ and σ).

**

Figure S 16: Pearson correlation coefficients for parameter combinations specified in the title, with changing interaction parameters (ρ and σ).

**

Figure S 17: Pearson correlation coefficients for parameter combinations specified in the title, with changing interaction parameters (ρ and σ).

**

Figure S 18: Pearson correlation coefficients for parameter combinations specified in the title, with changing interaction parameters (ρ and σ).

**

Figure S 19: Pearson correlation coefficients for parameter combinations specified in the title, with changing interaction parameters (ρ and σ).

**

Figure S 20: Pearson correlation coefficients for parameter combinations specified in the title, with changing interaction parameters (ρ and σ).

**

Figure S 21: Pearson correlation coefficients for parameter combinations specified in the title, with changing interaction parameters (ρ and σ).

**

Figure S 22: Pearson correlation coefficients for parameter combinations specified in the title, with changing interaction parameters (ρ and σ).

**

Figure S 23: Pearson correlation coefficients for parameter combinations specified in the title, with changing interaction parameters (ρ and σ).

Figure S 24: Pearson correlation coefficients for parameter combinations specified in the title, with changing interaction parameters (ρ and σ).

Figure S 25: Pearson correlation coefficients for parameter combinations specified in the title, with changing interaction parameters (ρ and σ).

Figure S 26: Pearson correlation coefficients for parameter combinations specified in the title, with changing interaction parameters (ρ and σ).

1. *Convergence*

For each simulation, we generated 5 replicates, for which we ran two chains with 450 000 iterations as burn in followed by a further 250 000 iterations. For chains that did not converge, we extended the chains for a further 250 000 iterations iteratively until convergence was reached or a total of 1 200 000 iterations were run. Figure S 25 shows the number of runs that reached convergence for each parameter combination. We expect that all runs will converge with sufficient iterations, however we were faced with computational limits.

Figure S 27: Number of Simulations for which the mcmc chains converged.

1. *Individual Simulation Analysis*

**

Figure S 28: Inference results for simulation with $\sigma$ = 0.99 and $\rho$ = 0.1. A: Posterior densities for all estimate parameters. The black vertical line represents the simulated (true) value. B: Points – simulated weekly incidence from the simulation, by virus and age group. Lines represent the model fit using the median value from the posterior distribution for each parameter. Ribbons represent 95% quantiles of the model fit (Cis) C: Pearson correlation coefficient between each parameter combination in the model.

**

Figure S 29: Inference results for simulation with $\sigma$ = 0.1 and $\rho$ = 0.1. See the legend of Figure S3 for more details.

**

Figure S 30: Inference results for simulation with $\sigma$ = 0.8 and $\rho$ = 0.1. See the legend of Figure S3 for more details.

**

Figure S 31: Inference results for simulation with $\sigma$ = 0.7 and $\rho$ = 0.1. See the legend of Figure S3 for more details.

**

Figure S 32: Inference results for simulation with $\sigma$ = 0.6 and $\rho$ = 0.1. See the legend of Figure S3 for more details.

**

Figure S 33: Inference results for simulation with $\sigma$ = 0.5 and $\rho$ = 0.1. See the legend of Figure S3 for more details.

**

Figure S 34: Inference results for simulation with σ = 0.4 and ρ = 0.1. See the legend of Figure S3 for more details.

**

Figure S 35: Inference results for simulation with $\sigma$ = 0.3 and $\rho$ = 0.1. See the legend of Figure S3 for more details.

**

Figure S 36: Inference results for simulation with σ = 0.2 and ρ = 0.1. See the legend of Figure S3 for more details.

**

Figure S 37: Inference results for simulation with σ = 0.1 and ρ = 0.1. See the legend of Figure S3 for more details.

**

Figure S 38: Inference results for simulation with σ = 0.01 and ρ = 0.1. See the legend of Figure S3 for more details.

**

Figure S 39: Inference results for simulation with σ = 0.99 and ρ = 0.05. See the legend of Figure S3 for more details.

**

Figure S 40: Inference results for simulation with σ = 0.9 and ρ = 0.05. See the legend of Figure S3 for more details.

**

Figure S 41: Inference results for simulation with σ = 0.8 and ρ = 0.05. See the legend of Figure S3 for more details.

**

Figure S 42: Inference results for simulation with σ = 0.7 and ρ = 0.05. See the legend of Figure S3 for more details.

**

Figure S 43: Inference results for simulation with σ = 0.6 and ρ = 0.05. See the legend of Figure S3 for more details.

**

Figure S 44: Inference results for simulation with σ = 0.5 and ρ = 0.05. See the legend of Figure S3 for more details.

**

Figure S 45: Inference results for simulation with σ = 0.4 and ρ = 0.05. See the legend of Figure S3 for more details.

**

Figure S 46: Inference results for simulation with σ = 0.3 and ρ = 0.05. See the legend of Figure S3 for more details.

**

Figure S 47: Inference results for simulation with σ = 0.2 and ρ = 0.05. See the legend of Figure S3 for more details.

**

Figure S 48: Inference results for simulation with σ = 0.1 and ρ = 0.05. See the legend of Figure S3 for more details.

**

Figure S 49: Inference results for simulation with σ = 0.01 and ρ = 0.05. See the legend of Figure S3 for more details.

**

Figure S 50: Inference results for simulation with σ = 0.99 and ρ = 0.5. See the legend of Figure S3 for more details.

**

Figure S 51: Inference results for simulation with σ = 0.9 and ρ = 0.5. See the legend of Figure S3 for more details.

**

Figure S 52: Inference results for simulation with σ = 0.8 and ρ = 0.5. See the legend of Figure S3 for more details.

**

Figure S 53: Inference results for simulation with σ = 0.7 and ρ = 0.5. See the legend of Figure S3 for more details.

**

Figure S 54: Inference results for simulation with σ = 0.6 and ρ = 0.5. See the legend of Figure S3 for more details.

**

Figure S 55: Inference results for simulation with σ = 0.5 and ρ = 0.5. See the legend of Figure S3 for more details.

Figure S 56: Inference results for simulation with σ = 0.4 and ρ = 0.5. See the legend of Figure S3 for more details.

**

Figure S 57: Inference results for simulation with σ = 0.3 and ρ = 0.5. See the legend of Figure S3 for more details.

**

Figure S 58: Inference results for simulation with σ = 0.2 and ρ = 0.5. See the legend of Figure S3 for more details.

**

Figure S 59: Inference results for simulation with σ = 0.1 and ρ = 0.5. See the legend of Figure S3 for more details.

**

Figure S 60: Inference results for simulation with σ = 0.01 and ρ = 0.5. See the legend of Figure S3 for more details.

**

Figure S 61: Inference results for simulation with σ = 0.99 and ρ = 0.2. See the legend of Figure S3 for more details.

**

Figure S 62: Inference results for simulation with σ = 0.9 and ρ = 0.2. See the legend of Figure S3 for more details.

**

Figure S 63: Inference results for simulation with σ = 0.8 and ρ = 0.2. See the legend of Figure S3 for more details.

**

Figure S 64: Inference results for simulation with σ = 0.7 and ρ = 0.2. See the legend of Figure S3 for more details.

**

Figure S 65: Inference results for simulation with σ = 0.6 and ρ = 0.2. See the legend of Figure S3 for more details.

**

Figure S 66: Inference results for simulation with σ = 0.5 and ρ = 0.2. See the legend of Figure S3 for more details.

**

Figure S 67: Inference results for simulation with σ = 0.4 and ρ = 0.2. See the legend of Figure S3 for more details.

**

Figure S 68: Inference results for simulation with σ = 0.3 and ρ = 0.2. See the legend of Figure S3 for more details.

**

Figure S 69: Inference results for simulation with σ = 0.2 and ρ = 0.2. See the legend of Figure S3 for more details.

**

Figure S 70: Inference results for simulation with σ = 0.1 and ρ = 0.2. See the legend of Figure S3 for more details.

**

Figure S 71: Inference results for simulation with σ = 0.01 and ρ = 0.2. See the legend of Figure S3 for more details.

**

Figure S 72: Inference results for simulation with σ = 0.99 and ρ = 0.025. See the legend of Figure S3 for more details.

**

Figure S 73: Inference results for simulation with σ = 0.9 and ρ = 0.025. See the legend of Figure S3 for more details.

**

Figure S 74: Inference results for simulation with σ = 0.8 and ρ = 0.025. See the legend of Figure S3 for more details.

**

Figure S 75: Inference results for simulation with σ = 073 and ρ = 0.025. See the legend of Figure S3 for more details.

**

Figure S 76: Inference results for simulation with σ = 0.6 and ρ = 0.025. See the legend of Figure S3 for more details.

**

Figure S 77: Inference results for simulation with σ = 0.5 and ρ = 0.025. See the legend of Figure S3 for more details.

**

Figure S 78: Inference results for simulation with σ = 0.4 and ρ = 0.025. See the legend of Figure S3 for more details.

**

Figure S 79: Inference results for simulation with σ = 0.3 and ρ = 0.025. See the legend of Figure S3 for more details.

**

Figure S 80: Inference results for simulation with σ = 0.2 and ρ = 0.025. See the legend of Figure S3 for more details.

**

Figure S 81: Inference results for simulation with σ = 0.1 and ρ = 0.025. See the legend of Figure S3 for more details.

**

Figure S 82: Inference results for simulation with σ = 0.01 and ρ = 0.025. See the legend of Figure S3 for more details.

1. *Varying the R_0_*

The R_0_ value we used for influenza was 2.91. This led to reasonable epidemics and an R_effective_ at the start of the simulated season of 1.55. Some previous estimates have placed R_0_ for influenza below 2. We therefore modelled 5 additional scenarios, all with an R_0_ of 1.98.

1. Original parameters (R_0_ 2.91, R_effective_ 1.55 )
2. Reduced R_0_ (1.98, R_effective_ 1.06)
3. Reduced R_0_ (1.98), increased importation (125 cases per day, R_effective_ 1.06)
4. Reduced R_0_ (1.98, R_effective_ 1.09), increased importation (125 cases per day), increased influenza susceptibility (3% increased, upper bound of the confidence intervals for susceptibility that we used from Baguelin et al. 2013)
5. Reduced R_0_ (1.98, R_effective_ 1.26), increased importation (125 cases per day), increased influenza susceptibility (20% increased)
6. Reduced R_0_ (1.98, R_effective_ 1.26), increased importation (5000 cases per day), increased influenza susceptibility (20% increased)

These simulations, along with the simulations for the original parameter values (R_0_ = 2.91), are shown Figures S83 – S87 for five combinations of parameter sets.

In none of the scenarios other than the original were we able to replicate the influenza epidemic. In all additional scenarios, influenza peak incidence was lower and the duration of the epidemics was longer than a typical influenza season in the UK. While an increased amount of importations allowed the peak to shift forward the width and peak height in the additional scenarios indicated an R_0_ that in combination with the observed susceptibility was not compatible in the model with the shape of a typical influenza season in the UK. The typical influenza season in the UK lasts less than 2.5 months/10 weeks^6,7^ and occurs after/during the RSV epidemic^8^, so these simulations were not deemed appropriate.

Figure S 83: Simulations with different model parameters, for Simulation 23 (σ = 0.99, 1/ρ = 2 days). Model types are 1 – original parameter values with and R_0_ of 2.91. 2 – R_0_ of 1.98. 3 – R0 of 1.98 and increased importation (125 imports per day) , 4 – R_0_ of 1.98, increased importation (125 imports per day) and 3% increased susceptibility. 5 – R_0_ of 1.98, increased importation (125 imports per day) and 20% increased susceptibility. 6 – R_0_ of 1.98, increased importation (5000 imports per day) and 20% increased susceptibility.

Figure S 84: Simulations with different model parameters, for Simulation 30 (σ = 0.7, 1/ρ = 2 days). Model types are 1 – original parameter values with and R_0_ of 2.91. 2 – R_0_ of 1.98. 3 – R0 of 1.98 and increased importation (125 imports per day) , 4 – R_0_ of 1.98, increased importation (125 imports per day) and 3% increased susceptibility. 5 – R_0_ of 1.98, increased importation (125 imports per day) and 20% increased susceptibility. 6 – R_0_ of 1.98, increased importation (5000 imports per day) and 20% increased susceptibility.

Figure S 85: Simulations with different model parameters, for Simulation 32 (σ = 0.1, 1/ρ = 2 days). Model types are 1 – original parameter values with and R_0_ of 2.91. 2 – R_0_ of 1.98. 3 – R0 of 1.98 and increased importation (125 imports per day) , 4 – R_0_ of 1.98, increased importation (125 imports per day) and 3% increased susceptibility. 5 – R_0_ of 1.98, increased importation (125 imports per day) and 20% increased susceptibility. 6 – R_0_ of 1.98, increased importation (5000 imports per day) and 20% increased susceptibility.

Figure S 86: Simulations with different model parameters, for Simulation 41 (σ = 0.3, 1/ρ = 10 days). Model types are 1 – original parameter values with and R_0_ of 2.91. 2 – R_0_ of 1.98. 3 – R0 of 1.98 and increased importation (125 imports per day) , 4 – R_0_ of 1.98, increased importation (125 imports per day) and 3% increased susceptibility. 5 – R_0_ of 1.98, increased importation (125 imports per day) and 20% increased susceptibility. 6 – R_0_ of 1.98, increased importation (5000 imports per day) and 20% increased susceptibility.

Figure S 87: Simulations with different model parameters, for Simulation 49 (σ = 0.6, 1/ρ = 40 days). Model types are 1 – original parameter values with and R_0_ of 2.91. 2 – R_0_ of 1.98. 3 – R0 of 1.98 and increased importation (125 imports per day) , 4 – R_0_ of 1.98, increased importation (125 imports per day) and 3% increased susceptibility. 5 – R_0_ of 1.98, increased importation (125 imports per day) and 20% increased susceptibility. 6 – R_0_ of 1.98, increased importation (5000 imports per day) and 20% increased susceptibility.

1. *References*

1. Diekmann, O., Heesterbeek, J. A. P. & Roberts, M. G. The construction of next-generation matrices for compartmental epidemic models. *J. R. Soc. Interface* **7**, 873–85 (2010).

2. Henderson, F. W., Collier, A. M., Clyde, W. A. & Denny, F. W. Respiratory-Syncytial-Virus Infections, Reinfections and Immunity. *N. Engl. J. Med.* **300**, 530–534 (1979).

3. Glezen, W. P., Taber, L. H., Frank, A. L. & Kasel, J. A. Risk of primary infection and reinfection with respiratory syncytial virus. *Am. J. Dis. Child.* **140**, 543–6 (1986).

4. Reeves, R. M. *et al.* Estimating the burden of respiratory syncytial virus (RSV) on respiratory hospital admissions in children less than five years of age in England, 2007-2012. *Influenza Other Respi. Viruses* **11**, 122–129 (2017).

5. Ohuma, E. O. *et al.* The natural history of respiratory syncytial virus in a birth cohort: the influence of age and previous infection on reinfection and disease. *Am. J. Epidemiol.* **176**, 794–802 (2012).

6. Fleming, D. M. & Elliot, A. J. Lessons from 40 years’ surveillance of influenza in England and Wales. *Epidemiology and Infection* **136**, 866–875 (2008).

7. Jick, H. & Hagberg, K. W. Effectiveness of influenza vaccination in the United Kingdom, 1996-2007. *Pharmacotherapy* **30**, 1199–1206 (2010).

8. Gov.uk. Six major respiratory viruses reported from PHE and NHS laboratories (SGSS) in England and Wales between weeks 01/2007 and 44/201. Available at: https://www.gov.uk/government/uploads/system/uploads/attachment_data/file/639687/six_pathogens_-_Jan2007-Aug2017.pdf. (Accessed: 6th October 2017)
